# Laccase–TEMPO as an Efficient System for Doxorubicin Removal from Wastewaters

**DOI:** 10.3390/ijerph19116645

**Published:** 2022-05-29

**Authors:** Luiza Izabela Jinga, Madalina Tudose, Petre Ionita

**Affiliations:** 1 Department of Organic Chemistry, Biochemistry and Catalysis, Faculty of Chemistry, University of Bucharest, 90 Panduri, 050663 Bucharest, Romania; izabela.jinga@inflpr.ro; 2 National Institute for Lasers, Plasma and Radiation Physics, Atomistilor 409, 077125 Magurele, Romania; 3 Institute of Physical Chemistry, 202 Spl. Independentei, 060021 Bucharest, Romania; madalina_tudose2000@yahoo.com

**Keywords:** laccase, TEMPO free radical, doxorubicin, enzymatic degradation, catalyst, bioremediation, pollutant removal

## Abstract

A large number of drugs are used to treat different diseases, and thus to improve the quality of life for humans. These represent a real ecological threat, as they end up in soil or ground waters in amounts that can affect the environment. Among these drugs, doxorubicin is a highly cytotoxic compound used as anticancer medicine. Doxorubicin can be efficiently removed from wastewater or polluted water using a simple enzymatic (biocatalytic) system, employing the oxidoreductase enzyme laccase and a stable organic nitroxide-free radical, TEMPO. Results presented in this work (as percentage of removal) were obtained at pH 5 and 7, after 2, 4, 6, and 24 h, using different ratios between doxorubicin, laccase, and TEMPO. It was shown that longer time, as well as an increased amount of catalyst, led to a higher percentage of removal, up to 100%. The influence of all these parameters is also discussed. In this way it was shown that the laccase–TEMPO biocatalytic system is highly efficient in the removal of the anticancer drug doxorubicin from wastewaters.

## 1. Introduction

Doxorubicin is an important anticancer medicine used for treatment of different types of maladies [1]. It is a natural compound isolated firstly from a *Streptomyces* bacteria [2], which contains a hydrophobic anthraquinone ring and a hydrophilic amino sugar fragment [3]. This important drug has multiple mechanisms of action that include DNA intercalation, topoisomerase II inhibition, and so on; another mechanism of action of doxorubicin as a strong chemotherapeutic agent involves the formation of reactive oxygen species (ROS) [4,5]. Doxorubicin can also mediate the intracellular generation of some types of free radicals [6].

**Figure 1:**
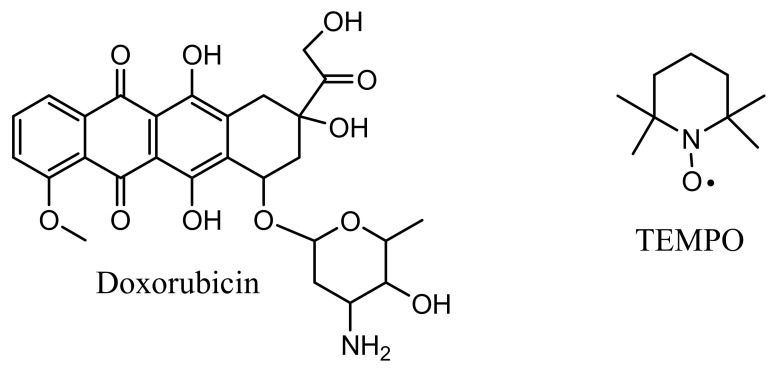
Chemical structure of doxorubicin and the TEMPO stable free radical.

However, doxorubicin treatments in humans are associated with high risks [7], and some synthetic derivatives were obtained in order to overcome such issues [8], preserving its therapeutic effect against tumor cells but reducing its toxicity to normal ones. Doxorubicin has a short half-life in the blood [9] and about half is excreted. The biodegradability of doxorubicin or other medicinal drugs (i.e., antibiotics) from wastewaters can be difficult and might have long-lasting negative effects. In the specific case of doxorubicin (Figure 1), the degradation kinetics in water have been well studied [10].

A highly effective and specific method to remove organic contaminants from polluted environments (and of course lowering their associated toxicity) is enzymatic treatment, which also offers the potential for end-user applications for an effective, greener pollution control [11,12]. Laccase [13] is a copper-containing polyphenol oxidase enzyme, found in many fungi, plants, and bacteria, which can oxidize a variety of compounds such as amines, phenols, etc., and some dyes and medicines, using molecular oxygen as final oxidant [14]. Besides, laccase is affordable and cheap in comparison with other reported metal complexes, and its efficiency in water as a natural environment may be desirable when dealing with hydrophobic derivatives [15]. Bacterial laccases are also involved in toxin oxidation or protection against oxidizing agents [16].

With all these benefits, laccase by itself can be used directly with very few substrates, but the introduction of laccase-mediator systems to overcome this limitation of its redox potential has led finally to extended applications, and therefore the mechanistic way of action is nowadays well-known [17].

The action of laccase substrates can be enlarged to higher-redox potential compounds that laccase itself cannot oxidize, with the help of a redox mediator, which can be a free radical [18]. For example, laccases are able to oxidize the TEMPO ((2,2,6,6-tetramethylpiperidin-1-yl)oxyl) stable free radical to the corresponding oxoammonium cation [19], a very strong oxidant that is further able to oxidize and decompose many organic compounds. For instance, laccase has been used for decolorization of azo dyes [20] and topical reviews are available [21].

Very recently [22], simple laccase was used for the removal of anticancer drugs from effluents, including doxorubicin (Figure 1). On the other hand, it is well-known that simple TEMPO can induce apoptosis of some cancer cells and suppress tumor growth [23], or have a protective effect in regard to doxorubicin cardiotoxicity [24].

Based on these considerations, the laccase/TEMPO/air system, used for the removal of doxorubicin via an oxidation/degradation process from wastewater or polluted water, can be regarded as a better, effective and greener approach, as no transition metals or other strong or harsh inorganic or organic oxidants are used.

In this work, we employed for the first time this laccase–TEMPO mediated system to study the process of removal of doxorubicin from water, varying and testing different working conditions in order to find the best settings that can be extended to an applicative process. Due to several advantages, doxorubicin was used as a model compound: (*i*) it is a natural chemical compound, intensely used in humans as medicine; (*ii*) it has a strong, bright red color, meaning that its concentration can be easily followed by simple techniques, such as UV–vis measurements; and (*iii*) it is also fluorescent, denoting a supplementary advantage in a possible dual-monitoring process and differentiation from other contaminants.

## 2. Materials and Methods

### 2.1. Chemicals

Doxorubicin hydrochloride was a product of AvaChem Scientific (Bucharest, Romania) and used as received. Laccase from *Trametes versicolor* was a Sigma product (Bucharest, Romania) with an activity of 0.84 U/mg. TEMPO stable free radical was from Acros Organics (Bucharest, Romania). Trisodium citrate dihydrate and citric acid were purchased from Roth (Bucharest, Romania). Double distilled purified water was used in all experiments. Chemicals were used as received and stored in proper conditions (4–10 °C).

### 2.2. Apparatus

UV–vis measurements were performed at 480 nm (the maximum wavelength of the doxorubicin) using either an Evolution 220 UV–vis spectrophotometer equipped with Insight software (Thermo Scientific, Schwerte, Germany) or a UVD-3500 double bean spectrophotometer (Labomed, LA, USA). Standard rectangular quartz cells with 0.5 or 1 cm optical path were used. A calibration curve was used for measuring the concentration of doxorubicin (see Figure S1, Supplementary Materials).

### 2.3. Methods

Stock solutions were prepared each day and kept in a fridge prior to measurements, with the following concentrations: TEMPO (1 mg/mL), laccase (1 mg/mL), and doxorubicin (0.1 mg/mL) were prepared in distilled water. Citrate buffers (0.1 M) were prepared by mixing 17.099 g of sodium citrate dihydrate and 8.042 g of citric acid in 800 mL distilled water. The solution pH was adjusted by using HCl or NaOH, and then distilled water was added until the final volume was 1 L.

In order to determine the degradation rate of doxorubicin in the presence of laccase and TEMPO, three batches of doxorubicin, with 0.5 mL, 1 mL, and 1.5 mL, were made. Each batch was then divided into five samples, by adding from stock solutions the same laccase and TEMPO volume, varying from 0.01 mL to 0.05 mL, then the citrate buffer was added to make a final volume of 2 mL. All samples were monitored at 0, 2, 4, 6, and 24 h.

The percentage of removal was calculated using the following formula:

$$\%\;of\;removal=\frac{\left[Dox0-Doxt\right]}{\left[Dox0\right]}\times 100$$

where *Dox*0 is the initial concentration of doxorubicin (at time 0) and *Dox*t is the concentration of doxorubicin at selected time (2, 4, 6, or 24 h).

## 3. Results and Discussion

**Figure 2:**
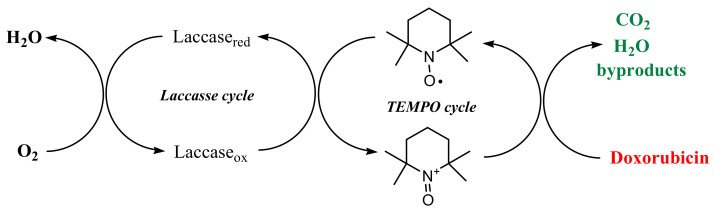
An overview of the laccase enzymatic process and of the TEMPO mediator in aerobic degradation of doxorubicin.

Doxorubicin is an organic compound that is intensely red colored (also fluorescent), and in the degradation process this bright color fades to colorless. Doxorubicin can be regarded as an anthraquinone dye, a polyphenol, and a saccharide derivative, and this makes it very susceptible to degradation processes following an oxidative path. Oxidation of organic compounds can be achieved employing high-oxidant transition metal cations (manganese and chromium derivatives being the most used), but this approach is considered very harmful for the environment, as these metal cations are extremely hard to remove from the system. As a greener approach, catalytic systems that avoid harsh chemicals can be used, with better results. The biocatalytic system of laccase/TEMPO (TEMPO being acting as a mediator) is well-known and has been reviewed many times in the literature [25], and nowadays is often used for the oxidation of phenols and amines via four single electron oxidation steps, using molecular oxygen from air [26]. Due to these important advantages, our work focused on employing such biocatalytic systems in the degradation of doxorubicin. The overall process is represented in Figure 2.

Both laccase and TEMPO work as catalysts in the activation process of oxygen. Thus, the oxidative form of the laccase enzyme transforms TEMPO free radical into an oxoammonium salt, a strong oxidant, which is able to degrade doxorubicin. In this process, TEMPO is also regenerated, while the reduced form of laccase is activated by the oxygen from air. The reaction mechanism of the laccase–TEMPO system is well documented in the literature [27].

Bioremediation using fungi-based technology (such as laccase) is regarded as a very cheap, effective, and environmentally friendly way of removing different pollutants, including human drugs from wastewaters [28]. Thus, this double catalyst process uses only oxygen from air as an oxidant and finally degrades doxorubicin, making the whole process effective without the use of any metal cations or other harsh chemicals that may further affect the environment.

Firstly, several tests were made, in order to achieve the best results and also to see if the proposed system works (and have some advantages over the literature data). From the literature data we know that the best pH working domain is between 5 and 7 [26,29], therefore the tests were performed in citrate buffers with standard pH values of 5 or 7. As doxorubicin slightly decomposes in the presence of laccase [22], we also tested first (*i*) its own degradation as a plain aqueous solution, (*ii*) its degradation in the presence of enzyme laccase, and (*iii*) its degradation in the presence of TEMPO free radical. Measurements were made at 2, 4, 6, and 24 h, and at pH 5 and 7, as already mentioned.

**Figure 3:**
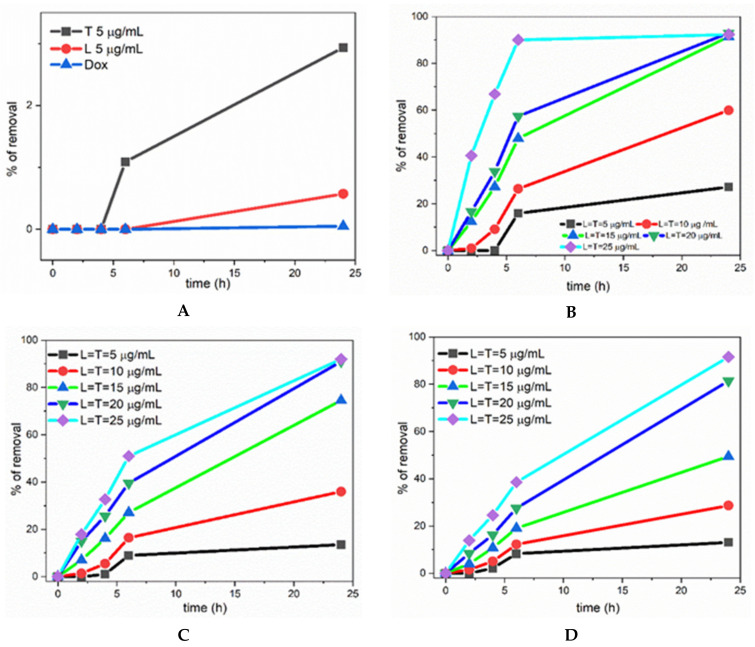
Percentage of removal of doxorubicin at pH 5: (**A**) blue, plain doxorubicin; red, in the presence of laccase; black, in the presence of TEMPO; (**B**–**D**) with the simultaneous presence of the laccase enzyme (L) and TEMPO (T) free radical. Starting doxorubicin concentrations: (**A**) 75 mg/mL; (**B**) 25 mg/mL; (**C**) 50 mg/mL; (**D**) 75 mg/mL.

**Figure 4:**
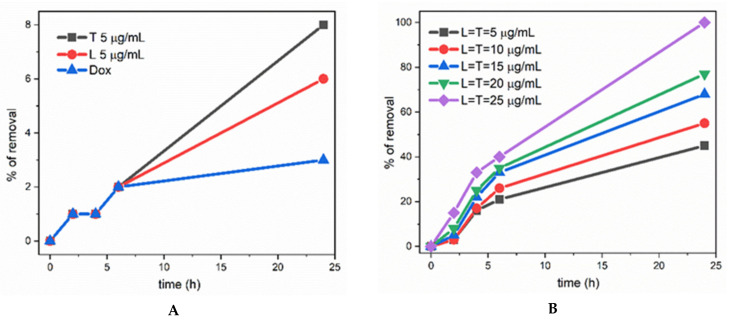

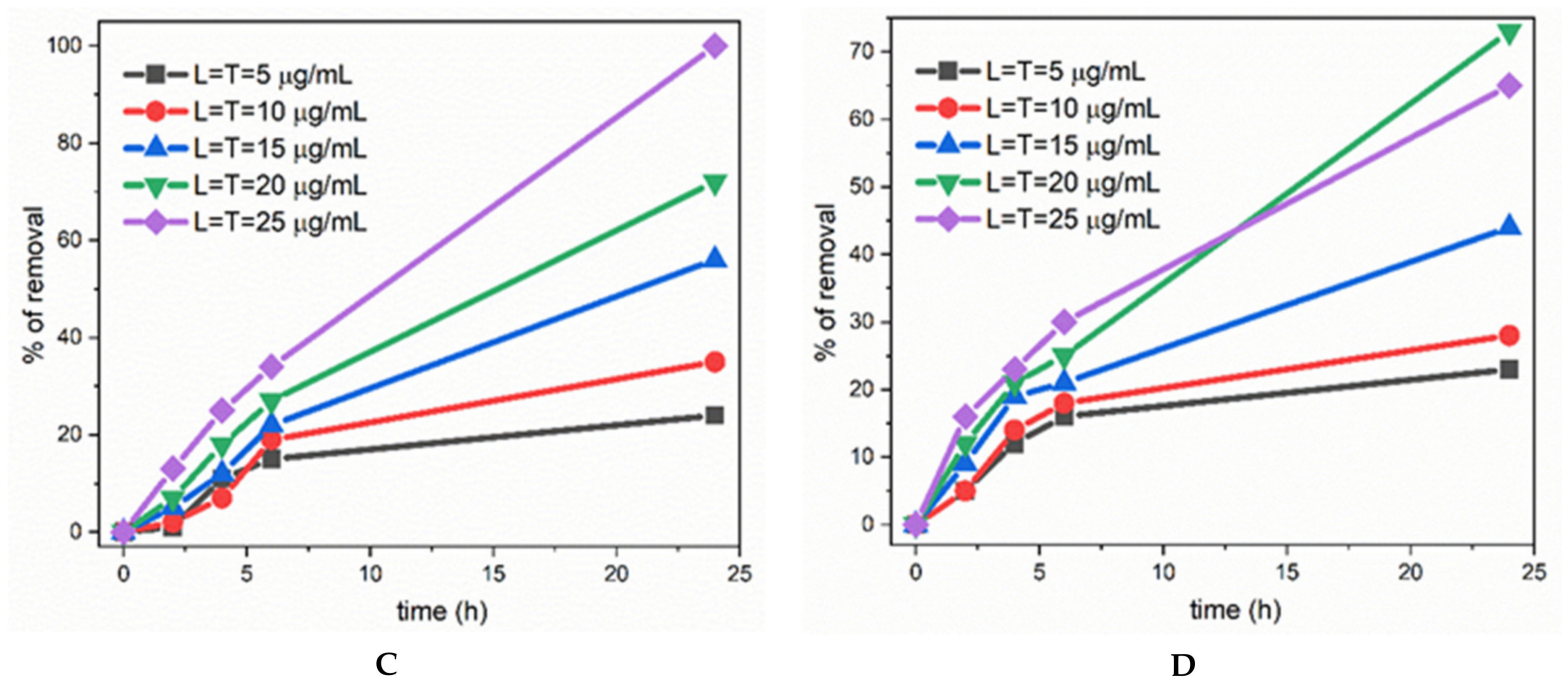
Percentage of removal of doxorubicin at pH 7: (**A**) blue, plain doxorubicin; red, in the presence of laccase; black, in the presence of TEMPO; (**B**–**D**) with the simultaneous presence of the laccase enzyme (L) and TEMPO (T) free radical. Starting doxorubicin concentrations: (**A**) 75 mg/mL; (**B**) 25 mg/mL; (**C**) 50 mg/mL; (**D**) 75 mg/mL.

Figure 3A shows the results obtained at pH 5, while Figure 4A shows the results obtained at pH 7. On its own, doxorubicin decomposition is very slow, reaching a maximum of 0–3% after 24 h, and in the presence of only laccase 1–6%; in the presence of TEMPO, again a low amount of doxorubicin decomposes, from 3–8% (Figure 3A and Figure 4A). These results mean that the employment of only one catalytic cycle (either the enzymatic cycle of laccase or the TEMPO free radical cycle, as depicted Figure 2) in the degradation of doxorubicin does not lead to a practical and effective result with regard to doxorubicin degradation, even after 24 h.

However, the simultaneous presence of laccase and TEMPO (considered a greener chemistry approach [29,30]) induces a dramatic change: as can be noted in Figure 3B–D and Figure 4B–D, the percentage of removal is close to 90–100% in some cases. Details will be discussed next.

The influence of the reaction time, of the concentrations, and of the ratios between the three components (doxorubicin, laccase, TEMPO) of the chosen system can be clearly seen following the trends in Figure 3 and Figure 4.

Thus, in all these pictures it is observed that: (*i*) a higher time of reaction led to higher yields of removal of doxorubicin; (*ii*) the rate of doxorubicin degradation was higher in the first 6 h; *(iii*) a higher ratio between the biocatalytic system (laccase/TEMPO) and the doxorubicin yielded also a higher percentage of removal.

In this way, in Figure 3B–D and Figure 4B–D it is noted that by increasing the concentration of TEMPO and laccase from 5 mg/mL to 25 mg/mL, the degradation percentage at 24 h rises from 10–20% to more than 90% (for exact values see Tables S1 and S2 from the Supplementary Materials).

Regarding the influence of the concentration of doxorubicin, and also the influence of different ratios between laccase and TEMPO on the final values of the percentage of removal, a different set of measurements were performed. Thus, we used next a different ratio between the concentration of the enzyme laccase and TEMPO free radical, following the same three concentrations of doxorubicin that were used before.

**Figure 5:**
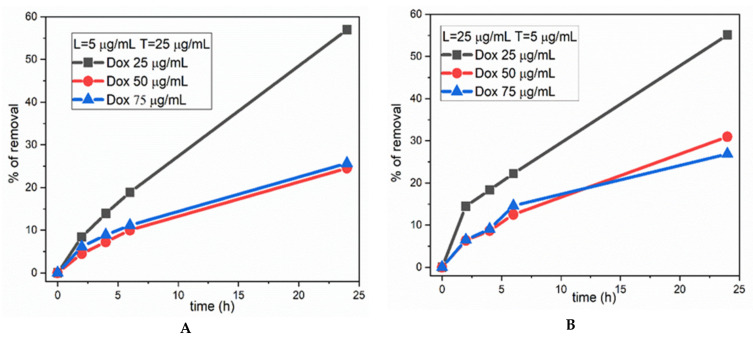
Percentage of removal of doxorubicin at different concentrations, varying the ratio between laccase and TEMPO: (**A**) laccase 5 mg/mL, TEMPO 25 mg/mL; (**B**) laccase 25 mg/mL, TEMPO 5 mg/mL; the concentrations of doxorubicin were 25, 50, and 75 mg/mL.

Figure 5 shows the data obtained using doxorubicin at three different concentrations, 25 mg/mL (red), 50 mg/mL (blue), and 75 mg/mL (black). The percentage of removal measured using different ratios between the enzyme laccase and TEMPO free radical was slightly smaller (by a few percentage points) when the concentration of laccase was 5 mg/mL and the concentration of TEMPO was 25 mg/mL (Figure 5A), compared with the case in which the concentration of laccase was 25 mg/mL and the concentration of TEMPO was 5 mg/mL (Figure 5B). As a partial conclusion, these parameters seem to have a small influence on the final percentage of removal, and also are not affected by the time of reaction. A similar conclusion can be drawn about the influence of pH.

Although in this work no attempts to recycle the laccase and TEMPO were made (the employed microgram concentrations cannot be successfully isolated for reuse), further work on immobilization of laccase and/or TEMPO on inert materials can be achieved, for a better reusability of the catalyst [31,32,33]. Anyway, the process can be extended for removal of other synthetic dyes [34], so the use of this specific dual system of laccase–TEMPO can be regarded as a multipurpose biocatalyst for environmental remediation [18].

## 4. Conclusions

The system employed in this study, bringing together the laccase enzyme as a multipurpose biocatalyst and TEMPO stable free radical as a redox mediator, showed that it is able to remove doxorubicin from wastewater, constituting a greener approach for medical or pharmaceutical environmental remediation. This biocatalytic process can be easily extended to suitable substrates, including other medicinal drugs, dyes, organic contaminants, and so on.

## Data Availability

Not applicable.

## Supplementary Materials

The following supporting information can be downloaded at: https://www.mdpi.com/article/10.3390/ijerph19116645/s1, Table S1. Influence of time and concentrations of doxorubicin, TEMPO and laccase upon the % of removal of doxorubicin at pH 5. Table S2. Influence of time and concentrations of doxorubicin, TEMPO and laccase upon the % of removal of doxorubicin at pH 7. Figure S1. Calibration curve showing the correlation of the doxorubicin concentration with the values of the registered absorbance at 480 nm.
