# Supplementary material for: Laccase–TEMPO as an Efficient System for Doxorubicin Removal from Wastewaters
Source: Int J Environ Res Public Health. 2022 May 29;19(11):6645. doi: 10.3390/ijerph19116645 (PMC9180534; doi:10.3390/ijerph19116645)

Laccase-TEMPO as an efficient system for doxorubicin removal from wastewaters

L.I. Jinga, M. Tudose, P. Ionita

Supplementary Material

Table S1. Influence of time and concentrations of doxorubicin, TEMPO and laccase upon the % of removal of doxorubicin at pH 5

laccase and TEMPO	doxorubicin											
	25 μg/mL				50 μg/mL				75 μg/mL			
	2 h	4 h	6 h	**24 h**	2 h	4 h	6 h	**24 h**	2 h	4 h	6 h	**24 h**
5 μg/mL	0	1	16	**27**	0	1	9	**14**	0	2	8	**13**
10 μg/mL	1	9	26	**60**	1	5	16	**36**	2	5	12	**29**
15 μg/mL	12	27	48	**91**	7	16	27	**75**	4	11	19	**49**
20 μg/mL	17	34	57	**92**	15	26	40	**91**	8	16	28	**81**
25 μg/mL	41	67	90	**93**	18	33	51	**92**	14	25	39	**92**

Table S2. Influence of time and concentrations of doxorubicin, TEMPO and laccase upon the % of removal of doxorubicin at pH 7

laccase and TEMPO	doxorubicin											
	25 μg/mL				50 μg/mL				75 μg/mL			
	2 h	4 h	6 h	**24 h**	2 h	4 h	6 h	**24 h**	2 h	4 h	6 h	**24 h**
5 μg/mL	3	16	21	**45**	1	11	15	**24**	5	12	16	**23**
10 μg/mL	3	17	26	**55**	2	7	19	**35**	5	14	18	**28**
15 μg/mL	5	22	33	**68**	5	12	22	**56**	9	19	21	**44**
20 μg/mL	8	25	35	**77**	7	18	27	**72**	12	21	25	**73**
25 μg/mL	15	33	40	**100**	13	25	34	**100**	16	23	30	**65**

Figure S1. Calibration curve showing the correlation of the doxorubicin concentration with the values of the registered absorbance at 480 nm

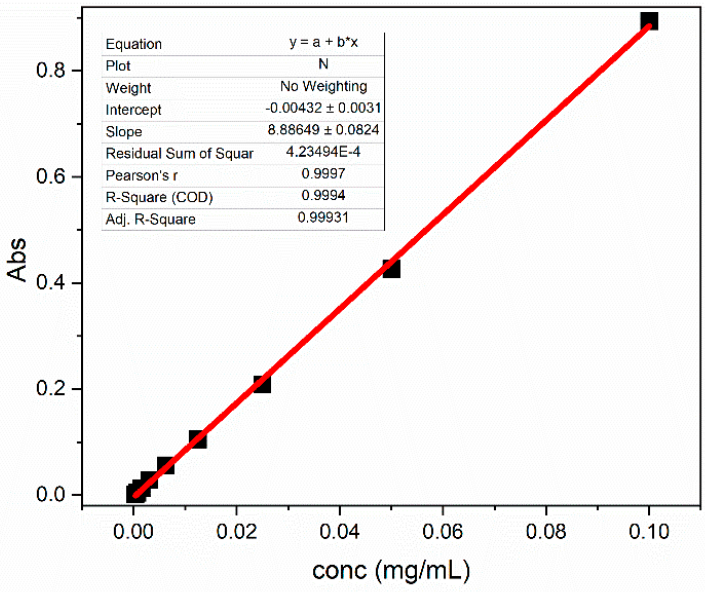

Supplement: Supplementary file 1 [file ijerph-19-06645-s001.zip › ijerph-1732510-supplementary.pdf]
